# A cross-sectional study on exposure to secondhand smoke in indoor public places and attitudes of residents towards the smoke control ordinance in public places

**DOI:** 10.18332/tid/196676

**Published:** 2024-12-27

**Authors:** Xiaowen Wang, Wenjing Zou, Xinyue Zhang, Nongnong Yang, Yuying Zheng, Runtang Meng, Haiyan Ma

**Affiliations:** 1 School of Public Health, Hangzhou Normal University, Hangzhou, China; 2 Wenzhou Center for Disease Prevention and Control, Wenzhou, China

**Keywords:** SHS, public places, tobacco control, attitudes, satisfaction

## Abstract

**INTRODUCTION:**

Secondhand smoke (SHS) exposure is a serious public health problem. This study aims to collect data on tobacco control since the implementation of the new version of the Smoke Control Ordinance in Public Places of Hangzhou (the Ordinance), combined with questionnaire surveys to understand the SHS exposure situation in public places, relevant knowledge and attitudes of residents in Hangzhou.

**METHODS:**

We used data from a population-based tobacco control survey of 2746 adults aged 15–75 years conducted in Hangzhou, Zhejiang Province in March 2023. Data were collected using a self-administered questionnaire consisting of demographic characteristics, exposure to tobacco, awareness of tobacco hazards, knowledge of the Ordinance, and behaviors and attitudes towards tobacco control in public places. Chi-squared tests were performed to examine the differences in knowledge and attitudes related to tobacco control in indoor public places among residents with different characteristics in Hangzhou. Multivariable logistic regression was used to identify variables affecting satisfaction with the effectiveness of tobacco control in public places.

**RESULTS:**

Of the 2155 non-smokers, 1006 (46.68%) had been exposed to SHS in public places. More than 89.00% of participants supported the Ordinance. Satisfaction with effectiveness of tobacco control in public places in Hangzhou was 68.54%, and the subcomponents that influenced it were satisfaction with tobacco control publicity campaigns (AOR=1.85; 95% CI: 1.19–2.88), satisfaction with tobacco control surveillance and enforcement (AOR=3.91; 95% CI: 2.43–6.30), satisfaction with the smoke-free demonstration for government departments (AOR=5.79; 95% CI: 3.96–8.47), and satisfaction with individual tobacco control behavior (AOR=11.68; 95% CI: 8.53–15.99).

**CONCLUSIONS:**

It is necessary to strengthen tobacco control publicity to increase the participation of residents and to gain a deeper understanding of the subjective willingness and needs of residents to participate in tobacco control campaigns in order to improve residents' individual satisfaction.

## INTRODUCTION

Tobacco smoke is one of the leading causes of death worldwide. It affects the health of the smokers and it is a threat to the lives of people inhaling secondhand smoke^1^. Every year, approximately 7 million people die from diseases caused by smoking, of which approximately 1.2 million die from diseases caused by exposure to secondhand smoke^2^.

Secondhand smoke (SHS) exposure comes from the inhalation of smoke from burning cigarettes, cigars, and pipes^3^. SHS contains chemicals that contribute to environmental pollution and many studies have clearly linked SHS exposure to adverse health consequences in non-smokers, including lung cancer, heart disease and asthma in children^4^. Despite these grave consequences, a large percentage of the world is still exposed to SHS. In 2004, disability-adjusted life-years (DALYs) loss caused by exposure to secondhand smoke reached 10.9 million, accounting for approximately 0.7% of the global burden of DALY diseases, and 61.00% of DALYs occurring in children^5^. It was estimated that globally about one-fifth of males and one-third of women were exposed to SHS and that this exposure caused almost 900000 deaths in 2016^6^. All this evidence suggests that expanding effective public health interventions to reduce SHS exposure can reduce the incidence of many adverse health consequences and diseases, and also reduce the burden on families and society. In order to ameliorate the various burdens caused by tobacco, a series of laws and regulations have been implemented globally. WHO’s Framework Convention on Tobacco Control (FCTC), ratified in 2005 by 168 countries that were signatories and 182 parties in which it was legally binding^7^, aimed to provide guidelines to implement effective tobacco control measures. Implementation of the Framework Convention on Tobacco Control has led to a significant reduction in smoking prevalence and associated hazardous consequences. The MPOWER policy package was introduced in 2008 and consists of the following six evidence-based policy components to help countries implement the FCTC: Monitor tobacco use and prevention policies; Protect people from tobacco smoke; Offer to help quit tobacco use; Warn about the dangers of tobacco; Enforce bans on tobacco advertising, promotion and sponsorship; and Raise taxes on tobacco^8^.

China was the largest producer and consumer of tobacco products in the world, with about 300 million smokers and 740 million SHS victims^9^. The 2018 China Global Adult Tobacco Survey showed that the prevalence of SHS exposure among non-smokers is high (68.10%)^10^. Faced with this severe situation, China has taken a series of legislative measures. Hangzhou was one of the earliest cities in China to start smoking control. The Hangzhou Municipal People’s Government in China first introduced the Smoke Control Ordinance in Public Places of Hangzhou (the Ordinance) in 2010^11^. In 2019, the city amended the Ordinance to further regulate smoking in public places. These amendments included banning smoking in indoor public places, workplaces and public transportation, thus protecting the health of more than 12 million people. The ban also extended to the use of electronic cigarettes in public places where smoking is prohibited^11^.

This study aims to collect data on tobacco control since the implementation of the new version of the Ordinance in order to understand the SHS exposure situation in public places, relevant knowledge and attitudes of residents in Hangzhou from public surveys. We thus provide theoretical guidance for making future recommendations to improve the Ordinance.

## METHODS

### Study design, population, and sampling

We used data from a population-based tobacco control survey of adults aged 15–75 years conducted in Hangzhou, Zhejiang Province in March 2023. According to the geographical distribution of Hangzhou districts and counties, five districts and counties were selected. The questionnaire survey was conducted using a sampling method, with an interception survey targeting Hangzhou citizens who were observing the activities of the sampled places on site during the survey time period. The surveyed establishments were categorized into different types such as government departments, hospitals, training institutes, entertainment venues, restaurants, hotels and shopping malls according to the nature of their operation, totaling 26 establishments. At least 20 questionnaires were distributed to each type of establishment. The sample size for each type of establishment is given in Supplementary file Table 1. We used the sample size calculation formula^12^:


$$n=\frac{Z_{0.05}^{2}\times p\left(1-p\right)}{\delta^{2}}$$

where Z=1.96 when the confidence level is 95%, p is the probability, δ is the allowable error taken as p=0.15, giving a sample size of 456 for each district, totaling a sample size of 2280.

Individuals aged 15–75 years who were residents or have lived in the local area for at least 6 months were eligible to participate in the study. Individuals with mental disorders and those unable to co-operate with the investigation were excluded. The final sample size included in the study was 2746. The study protocol was approved by the Scientific Research Ethics Committee of Hangzhou Normal University (Approval number: 20190100; Date: October 20, 2020).

### Data collection

The survey used a uniformly designed questionnaire, coordinated and organized by the Hangzhou Tobacco Control Office. The questionnaire included demographic characteristics, individual smoking behavior and exposure to tobacco, awareness of tobacco hazards and knowledge of the Ordinance, support for and willingness to participate in tobacco control campaigns in public places, and satisfaction with tobacco control in public places (including satisfaction with tobacco control publicity campaigns, tobacco control surveillance and enforcement, the smoke-free demonstration for government departments, and individual tobacco control behavior).

Demographic characteristics included gender, age, residence, education level, occupation, marital status and chronic diseases. Individual smoking behavior and tobacco exposure were assessed by three questions: 1) ‘Do you use tobacco products, including cigarettes, hookahs or water pipes, medwakh (traditional Arabic pipes) and cigars?’; 2) ‘How many days in the last 7 days have you been exposed to tobacco in public places?’; and 3) ‘What indoor places have you been exposed to SHS in the last 7 days?’. Awareness of tobacco hazards was assessed by three questions: 1) ‘To your knowledge, can smoking cause stroke/heart attack/lung cancer?’; 2) ‘To your knowledge, can inhaling SHS cause adult heart disease/children’s lung disease/adult lung cancer?’; and 3) ‘To your knowledge, is thirdhand smoke a health hazard?’. Awareness of knowledge of the Ordinance was assessed by five questions: 1) ‘According to the Tobacco Control Ordinance, what else can law enforcement officers do to discourage illegal smokers in public places?’; 2) ‘What is the hotline number for tobacco control complaints in Hangzhou?’; 3) ‘To your knowledge, which of the following places does the current Ordinance prohibit smoking?’; 4) ‘To your knowledge, can a man aged 35 years get his son aged 15 years to take his ID card and buy cigarettes for him?’; and 5) ‘To your knowledge, are e-cigarettes allowed in public places in Hangzhou?’. Behaviors towards tobacco control in public places were assessed by three questions: 1) ‘Have you complained about smoking in public places in the last six months?’; 2) ‘Have you discouraged smoking in public places in the last six months?’; and 3) ‘Have you participated in any tobacco control campaigns in the last six months?’. Attitudes towards tobacco control in public places were assessed by eight questions: 1) ‘Would you be willing to call the complaint hotline if someone is smoking in a public place?’; 2) ‘Would you be willing to go up to someone and discourage them from smoking in a public place?’; 3) ‘Would you like to participate in a tobacco control campaign?’; 4) ‘Are you satisfied with tobacco control publicity campaigns in the past year?’; 5) ‘Are you satisfied with the tobacco control surveillance and enforcement in the past year?’; 6) ‘Are you satisfied with the smoke-free demonstration for government departments in the past year?’; 7) ‘Are you satisfied with your tobacco control behavior in public places in the past year?’; and 8) ‘Are you satisfied with the effectiveness of tobacco control in indoor public places in the city in the past year?’.

### Definition of variables

Tobacco use was defined as smoking >1 cigarette per day for >6 consecutive or cumulative months. SHS exposure was defined as the exposure of non-smokers to smoke emitted from the end of a lit cigarette or exhaled by a smoker for ≥1 day per week^7^. Exposure rate to SHS in public places was the proportion of non-smokers exposed to SHS in any public place, including indoor public places and indoor workplaces. Indoor public places were places where smoking was prohibited indoors as specified in the Ordinance, including public transportation, schools, medical institutions, sub-office halls, community hallways, hotels, restaurants, entertainment places and shopping malls where smoking was prohibited. The people covered by indoor work were indoor workers or the workplace had indoor areas; people involved in indoor public places did not include people in workplaces. Respondents’ awareness of tobacco hazards and knowledge of the Ordinance, were expressed as percentages. There were three questions related to the awareness of tobacco hazards, with a score of 7, and 4 was considered as meeting the standard. There were five questions related to the core knowledge of the Ordinance, with a score of 16, and 10 was considered as meeting the standard. Respondents’ support for the Ordinance was evaluated using a five-level categorization, and the choices of ‘very supportive’ and ‘relatively supportive’ were defined as supportive, and the choices of ‘general’, ‘oppose’ and ‘very oppose’ were defined as low willingness. A five-point Likert scale was used to evaluate respondents’ willingness to participate in tobacco control campaigns in public places and their satisfaction (5=very willing/very satisfied to 1=very unwilling/unsatisfied), in which scores of ≥4 were defined as high willingness, and scores <4 were defined as low willingness. In this study, according to the logical sequence of the implementation process of tobacco control regulations, the satisfaction with tobacco control was divided into satisfaction with tobacco control publicity campaigns, tobacco control surveillance and enforcement, the smoke-free demonstration for government departments, and individual tobacco control behavior. A reliability analysis of the questionnaire on behavior and attitudes towards tobacco control in public places showed that Cronbach’s alpha was 0.80, indicating that the questionnaire had a good reliability.

### Statistical analysis

All statistical analyses were performed using SPSS 22.0 (https://www.ibm.com/cn-zh/spss). The sample was described using frequencies and percentages for categorical variables, and means and standard deviations (SD) for continuous variables. Chi-squared tests were performed to examine the differences in knowledge and attitudes related to tobacco control in indoor public places among residents with different characteristics in Hangzhou. Differences in satisfaction with tobacco control in public places among residents of Hangzhou with different characteristics were also tested with chi-squared tests. Multivariable logistic regression was used to identify variables affecting satisfaction with the effectiveness of tobacco control in public places, from which adjusted odds ratios (AORs) and 95% confidence intervals (95% CIs) were calculated. We used the Wald test for testing the significance of explanatory variables. A p<0.05 (two-tailed) was considered statistically significant.

## RESULTS

### Demographic characteristics

A total of 2746 participants were interviewed in this survey; 1350 (49.16%) males and 1396 (50.84%) females. Of the sample, 2155 (78.48%) were non-smokers (including former smokers). General characteristics of the sample are shown in Table 1. Respondents were aged 15–75 years, with the highest percentages in the age groups of 15–25 and 26–35 years. The majority of respondents were married (55.43%) and located in urban areas (81.03%). According to self-reporting, the percentage of patients with chronic diseases was 11.03%.

**Table 1:** Comparison of knowledge and attitudes related to tobacco control in indoor public places among residents, Hangzhou, China, 2022 (N=2746)

*Characteristics*	*Total n (%)*	*Awareness*				*Willingness to support*	
		*Tobacco hazards n (%)*	*χ^2^*	*Knowledge of the Ordinance n (%)*	*χ^2^*	*n (%)*	*χ^2^*
**Gender**			30.53***		0.49		54.48***
Male	1350 (49.16)	769 (56.96)		895 (66.30)		1155 (85.55)	
Female	1396 (50.84)	938 (67.19)		943 (67.55)		1313 (94.05)	
**Age** (years)			146.18***		9.49		9.49**
15–25	807 (29.39)	565 (70.01)		524 (64.93)		719 (89.10)	
26–35	867 (31.57)	575 (66.32)		582 (67.13)		792 (91.35)	
36–45	492 (17.92)	323 (65.65)		344 (69.92)		453 (92.07)	
46–55	274 (9.98)	138 (50.36)		197 (71.90)		245 (89.42)	
56–65	206 (7.50)	76 (36.89)		127 (61.65)		177 (85.92)	
66–75	100 (3.64)	30 (30.00)		64 (64.00)		82 (82.00)	
**Residence**			13.67*		25.97***		16.00**
Urban	2225 (81.03)	1371 (61.62)		1503 (67.55)		1987 (89.30)	
Rural	521 (18.97)	336 (64.49		335 (64.30)		481 (92.32)	
**Education level**			199.52***		4.32		35.15***
Elementary school or lower	138 (5.03)	42 (30.43)		85 (61.59)		113 (81.88)	
Junior high school	422 (15.37)	194 (45.97)		284 (67.30)		356 (84.36)	
High school	602 (21.92)	321 (53.32)		390 (64.78)		536 (89.04)	
Junior college/college	1431 (52.11)	1040 (72.68)		972 (67.92)		1323 (92.45)	
Postgraduate or higher	153 (5.57)	110 (71.90)		107 (69.93)		140 (91.50)	
**Occupation**			120.96***		10.35		22.52***
Institutionalized personnel	251 (9.14)	185 (73.71)		177 (70.52)		236 (94.02)	
Business units	752 (27.39)	504 (67.02)		505 (67.15)		694 (92.29)	
Students	374 (13.62)	288 (77.01)		258 (68.98)		342 (91.44)	
Freelance	502 (18.28)	291 (57.97)		348 (69.32)		447 (89.04)	
Retired	198 (7.21)	80 (40.40)		134 (67.68)		171 (86.36)	
Other	669 (24.36)	359 (53.66)		416 (62.18)		578 (86.40)	
**Marital status**			47.81***		8.31*		10.82*
Single	1144 (41.66)	788 (68.88)		738 (64.51)		1024 (89.51)	
Married/cohabiting	1522 (55.43)	880 (57.82)		1051 (69.05)		1380 (90.67)	
Divorced/separated	59 (2.15)	34 (57.63)		38 (64.41)		46 (77.97)	
Widowed	21 (0.76)	5 (23.81)		11 (52.38)		18 (85.71)	
**Chronic diseases**			29.65***		2.75		9.58**
Yes	303 (11.03)	1562 (63.94)		1648 (67.46)		2211 (90.50)	
No	2443 (88.97)	145 (47.85)		190 (62.71)		257 (84.82)	
**Tobacco use**			58.37***		0.01		153.03***
No	1973 (71.85)	1309 (66.35)		1320 (66.90)		1856 (94.07)	
Yes	591 (21.52)	290 (49.07)		396 (67.01)		453 (76.65)	
Ever smoked	182 (6.63)	108 (59.34)		122 (67.03)		159 (87.36)	
**Overall**	2746 (100)	1707 (62.16)		1838 (66.93)		2468 (89.88)	

* p<0.05,

** p<0.01,

*** p<0.001.

### Self-reported rate of exposure to SHS

In public places, among the 2155 non-smokers, 1006 (46.68%) had been exposed to SHS. There were differences between the exposed and non-exposed groups in terms of gender, education level, and occupation (p<0.05) (Supplementary file Table 2). Among the exposed population , 359 (16.66%) had been exposed to SHS in indoor workplaces, 848 (39.35%) in indoor public places, of whom 153 (7.10%), 75 (3.48%), 37 (1.72%), 87 (4.04%), 474 (22.00%), 106 (4.92%), 401 (18.61%), 280 (12.99%), and 277 (12.85%) had been exposed to SHS in public transportation, schools, medical institutions, sub-office halls, community hallways, hotels, restaurants, entertainment places and shopping malls, respectively (Table 2).

**Table 2:** Self-reported rate of exposure to SHS in public places among non-smokers, Hangzhou, China, 2022 (N=2155)

*Location*	*n*	*%*
**Public places**	1006	46.68
**Indoor workplaces**	359	16.66
**Indoor public places** (no smoking places)	848	39.35
Public transportation	153	7.10
Schools	75	3.48
Medical institutions	37	1.72
Sub-office halls	87	4.04
Community hallways	474	22.00
Hotels	106	4.92
Restaurants	401	18.61
Entertainment places	280	12.99
Shopping malls	277	12.85

SHS: secondhand smoke.

### Comparison of knowledge, attitudes and satisfaction with tobacco control in public places

The awareness and support rates of residents for the Ordinance are shown in Table 1. About 62.16% of all participants were aware of the hazards of smoking and 66.93% of all participants were aware of the Ordinance. In addition, more than 89.00% of the participants supported for the Ordinance. There were differences in awareness of tobacco hazards by gender, age, place of residence, education level, occupation, marital status, self-reported chronic disease and tobacco use (p<0.05); and differences in knowledge of the Ordinance by place of residence and marital status (p<0.05). Meanwhile, attitudes towards the Ordinance varied in terms of willingness to support it by gender, age, place of residence, education level, occupation, marital status, self-reported chronic disease status and tobacco use (p<0.05). The satisfaction with tobacco control in public places is shown in Supplementary file Table 3. Satisfaction with effectiveness of tobacco control in public places in Hangzhou was 68.54%. There were differences in satisfaction with tobacco control publicity campaigns, individual tobacco control behavior and effectiveness of tobacco control in indoor public places in Hangzhou by age, education level, occupation and marital status (p<0.05); and differences in satisfaction with tobacco control surveillance and enforcement and the smoke-free demonstration for government departments by gender, age, education level, occupation and marital status (p<0.05).

### Multivariable analyses

In the multivariable model, we controlled for gender, age, residence, education level, occupation, marital status, chronic diseases, tobacco use, willingness to participate in tobacco control campaigns, awareness of tobacco hazards and knowledge of the Ordinance. The subcomponents that influenced satisfaction with the effectiveness of tobacco control in public places, were satisfaction with tobacco control publicity campaigns (AOR=1.85; 95% CI: 1.19–2.88), satisfaction with tobacco control surveillance and enforcement (AOR=3.91; 95% CI: 2.43–6.30), satisfaction with the smoke-free demonstration for government departments (AOR=5.79; 95% CI: 3.96–8.47), and satisfaction with individual tobacco control behavior (AOR=11.68; 95% CI: 8.53–15.99) (Table 3). Among them, the largest positive effect was satisfaction with personal tobacco control behavior (Table 3).

**Table 3:** Multivariable logistic regression analysis of satisfaction with the effectiveness of tobacco control in public places, Hangzhou, China, 2022 (N=2746)

*Variables*	*Reference*	*β*	*SE*	*Wald*	*p*	*AOR (95% CI)*
Satisfaction with tobacco control publicity campaigns	Low	0.62	0.23	7.36	<0.01	1.85 (1.19–2.88)
Satisfaction with tobacco control surveillance and enforcement	Low	1.36	0.24	31.39	<0.001	3.91 (2.43–6.30)
Satisfaction with the smoke-free demonstration for government departments	Low	1.76	0.19	82.02	<0.001	5.79 (3.96–8.47)
Satisfaction with individual tobacco control behavior	Low	2.46	0.16	235.06	<0.001	11.68 (8.53–15.99)

AOR: adjusted odds ratio; adjusted for gender, age, locality type, education level, occupation, marital status, chronic diseases, tobacco use, willingness to participate in tobacco control campaigns, awareness of tobacco hazards, and knowledge of the Ordinance.

## DISCUSSION

This was a large-scale study to investigate SHS exposure in public places and tobacco control-related knowledge and attitudes among residents in Hangzhou. This study contributed to the literature in the following ways. First, our study revealed that residents aged 15–75 years in Hangzhou had a smoking prevalence of 21.52% and an exposure rate to SHS in public places of 46.68%. According to a study, the exposure rate to SHS in public places in Hangzhou was 90.36% in 2018^13^. Analyzing the reasons for the decrease, it may be related to the increase in smoking cessation rate and the increase in awareness of tobacco hazards and SHS due to the implementation of the Ordinance. The 2015 China Adult Tobacco Survey (CATS)^14^ showed that the overall smoking prevalence among Chinese residents was 27.70%, and the exposure rate to SHS in indoor workplaces was 54.30%. Preliminary findings from the latest round of GATS China in 2018 indicated that smoking prevalence decrease to 26.60%^15^. The effectiveness of tobacco control in Hangzhou may be attributed to the implementation of various regulations in recent years. In addition, compared with other cities in China, the SHS exposure rate in indoor workplaces of Hangzhou residents was 16.66%, lower than that of Heilongjiang Province in 2017 (49.80%)^16^, Shanghai (17.30%) in 2018^17^, and Wuhan (24.20%) in 2019^18^. These indicate that Hangzhou has achieved certain results in promoting the implementation of the city’s tobacco control ordinance in recent years. This has laid a good foundation for achieving the requirement of ‘gradually realizing a total ban on smoking in indoor public places’ in the Healthy China 2030 Plan^19^. However, compared with developed countries such as Canada and the United Kingdom, China still has a gap in the effectiveness of tobacco control^2^.

Second, our study explored the awareness and support rates of Hangzhou residents for the new version of the Ordinance. Findings showed that over 60.00% of residents had a basic understanding of the Ordinance and the dangers of tobacco, and more than 89.00% of the participants supported the Ordinance. According to previous studies^20^, in 2013, 58.22% of smokers and 79.76% of non-smokers in Zhejiang Province, agreed that smoking should be banned in public places. This implied that with time, the overall tobacco control awareness level of Hangzhou residents was increasing. In addition, this study indicated that there were differences in knowledge of tobacco harms by gender, age, place of residence, education level, occupation, marital status, self-reported chronic diseases and tobacco use, which was consistent with previous studies^21^. Meanwhile, there were differences in support for the Ordinance among residents by gender, age, residence, education level, occupation, marital status, chronic diseases and tobacco use. Rose et al.^22-24^ found that female, urban dwellers, people with higher education, higher income, and non-smokers were more supportive of the local tobacco policies.

Third, this study also explored the factors influencing residents’ overall satisfaction with tobacco control in indoor public places. It was implied that satisfaction with tobacco control publicity campaigns, satisfaction with tobacco control surveillance and enforcement, satisfaction with the smoke-free demonstration for government departments and satisfaction with individual tobacco control behavior, were positively related to overall satisfaction. Numerous studies have reported that smoke-free laws may substantially reduce smoking prevalence^25-27^. From the viewpoint of social norms, the behavior of people was influenced by their perceptions of what was ‘normal’ or ‘typical’. Smoke-free laws may alter social norms and lead people to change their beliefs, awareness, attitudes, and practices concerning smoking^26^. Meanwhile, public education can be effective in raising public consciousness and changing unfavorable beliefs and attitudes concerning tobacco control^7^. In recent years, Hangzhou has focused on the introduction of various regulations and tobacco control publicity work, which has led to the conscious co-operation of residents with the relevant work and achieved good results, resulting in a high level of overall satisfaction. Interestingly, our study found that the subjective factor of satisfaction with individual tobacco control behavior contributed most to overall satisfaction with tobacco control in indoor public places in Hangzhou. It may be that because satisfaction was itself a subjective indicator, people’s own tobacco control behaviors are, to some extent, better indicators of the effectiveness of tobacco control, which may be explained by several contemporary theories of learning motivation. Motivation had been defined as the process whereby goal-directed activities are initiated and sustained. Within the history of the study of human motivation, a number of theoretical perspectives elevated cognitions and conditions pertaining to expectations for the future as well as perceptions of autonomy to prominent motivational positions. Social-cognitive theory emphasizes self-efficacy as the primary driver of motivated action, and also identifies cues that influence future self-efficacy and support self-regulated learning. Self-determination theory proposes that optimal performance results from actions motivated by intrinsic interests or by extrinsic values that have become integrated and internalized^28^. Therefore, a large part of the improvement of satisfaction with tobacco control in public places in Hangzhou depended on the improvement of residents’ intrinsic motivation. Currently, more than 10 cities have smoke-free movements, and nationwide campaigns have been conducted to ban smoking on university campuses and in hospitals^29^. At the same time, Hangzhou was in dire need of further development in organizing tobacco control campaigns.

Nevertheless, Hangzhou and even China still face huge challenges in controlling SHS exposure in public places. Numerous international studies have shown that smoke-free legislation can effectively protect people from SHS in public places^27,30^. Currently, there are no national-level tobacco control regulations in China, and promoting local legislation in various places is difficult. Smoke-free laws that clearly ban e-cigarettes in smoke-free areas have been implemented in only two cities, Nanning and Hangzhou^31^. There is also evidence that existing bans are not well implemented. Yang et al.^32^, for example, found that 37.20% of respondents in six Chinese cities reported yes to noticing things that encourage smoking in the previous six months. Hangzhou, Zhejiang Province, began implementing the Ordinance on 1 January 2019, with new regulations requiring a total ban on smoking indoors, making it the 19th city to promulgate local regulations on smoke-free environments, but there is still a gap in the province’s goal of banning smoking in public places. In the future, Hangzhou should speed up the legislative process of smoke-free public places in the province, with strict regulatory penalties and publicity of smoke-free indoor public places, to ensure a smoke-free environment by law^33^, and through the creation of health-promoting communities to promote healthy smoke-free families and achieve a ban on smoking in the home, in-depth in schools, hospitals, communities, and enterprises to carry out a variety of forms of tobacco hazards and refusal to SHS, and the use of a strong media action to disseminate the hazards of SHS health education^33^. Our research methods and results can provide a theoretical basis for other countries or cities to explore secondhand smoke exposure and policy evaluation.

### Strengths and limitations

This study comprehensively described the SHS exposure in public places and residents’ attitudes towards the Ordinance. At the same time, the study had a sufficient and representative sample size. Nevertheless, there were several limitations in our study. First, the cross-sectional design of the study did not allow us to make a causal inference, thus, results needed to be interpreted with that in mind. Second, data used in this study were based on self-report and thus were subject to misclassification bias. Third, degree or duration of exposure to SHS was not collected and, for instance, dose–response could not be explored. Fourth, the study area was limited to one city, with limited generalizability to other cities/countries.Finally, due to space constraints and multiple considerations, we did not test the interaction term (gender × subgroup) in this study. We plan to test the interaction term to explore potential differential effects in the future research.

## CONCLUSIONS

Tobacco control in public places in Hangzhou had been effective but still need to be further strengthened or sustained. In the future, it is necessary to strengthen tobacco control enforcement, especially in high-risk places for SHS exposure such as community hallways, restaurants and indoor workplaces; strengthen tobacco control publicity to increase the participation of residents; and gain a deeper understanding of the subjective willingness and needs of residents to participate in tobacco control campaigns in order to improve residents’ individual satisfaction.

## Supplementary Material



## Data Availability

The data supporting this research are available from the authors on reasonable request.

## References

[1] U.S. Office on Smoking and Health. The Health Consequences of Involuntary Exposure to Tobacco Smoke: A Report of the Surgeon General. U.S. Centers for Disease Control and Prevention; 2006. Accessed December 1, 2024. https://www.ncbi.nlm.nih.gov/books/NBK44324/20669524

[2] World Health Organization. WHO Report on the Global Tobacco Epidemic 2021: Addressing new and emerging products. Accessed December 1, 2024. https://iris.who.int/bitstream/handle/10665/343287/9789240032095-eng.pdf?sequence=1

[3] U.S. Department of Health and Human Services. Eliminating Tobacco-Related Disease and Death: Addressing Disparities: A Report of the Surgeon General. Accessed December 1, 2024. https://www.hhs.gov/sites/default/files/2024-sgr-tobacco-related-health-disparities-full-report.pdf 40373173

[4] Barnoya J, Glantz SA. Cardiovascular effects of secondhand smoke: nearly as large as smoking. Circulation. 2005;111(20):2684-2698. doi:10.1161/CIRCULATIONAHA.104.492215 15911719

[5] Oberg M, Jaakkola MS, Woodward A, Peruga A, Prüss-Ustün A. Worldwide burden of disease from exposure to secondhand smoke: a retrospective analysis of data from 192 countries. Lancet. 2011;377(9760):139-146. doi:10.1016/S0140-6736(10)61388-8 21112082

[6] Hoe C, Cohen JE, Yang T, et al. Association of cigarette production and tobacco retailer density on secondhand smoke exposure in urban China. Tob Control. 2022;31:e118-e125. doi:10.1136/tobaccocontrol-2021-056655 34230057 PMC9726971

[7] World Health Organization. WHO Framework Convention on Tobacco Control. Accessed December 1, 2024. https://www.who.int/europe/teams/tobacco/who-framework-convention-on-tobacco-control-(who-fctc)

[8] Anderson CL, Mons U, Winkler V. Global progress in tobacco control: the question of policy compliance. Glob Health Action. 2020;13(1):1844977. doi:10.1080/16549716.2020.1844977 33190609 PMC7671716

[9] World Health Organization. Tobacco: Tobacco in China. Accessed December 1, 2024. https://www.who.int/china/health-topics/tobacco

[10] China Centers for Disease Control and Prevention. Survey results of adult tobacco in China in 2018. Website in Chinese. Accessed December 1, 2024. http://news.sina.com.cn/c/2019-05-31/doc-ihvhiqay2684108.shtml

[11] World Health Organization. Three tobacco control champions recognized in the Western Pacific Region. Accessed December 1, 2024. https://www.who.int/westernpacific/news-room/feature-stories/item/three-tobacco-control-champions-recognized-in-the-western-pacific-region

[12] Zhang X. Current Situation and Countermeasures of tobacco exposure in Public Places in Hangzhou. In Chinese. 2019.

[13] Zhang XY, Tan RY, Xie L, et al. Comparative analysis on changes in tobacco exposure and residents’ attitudes toward tobacco control in public places in Hangzhou before and after the implementation of the New Regulation. Article in Chinese. Chinese Journal of Health Education. 2022;38(5):427-431. doi:10.16168/j.cnki.issn.1002-9982.2022.05.009

[14] Tobacco Control Resource Center. China Adult Tobacco Survey. CDC, 2015. Website in Chinese. Accessed December 1, 2024. https://cmsfiles.zhongkefu.com.cn/yankong/UploadFiles/2016-03/318/201603231215175500.pdf

[15] Guo H, Quan G. Tobacco control in China and the road to Healthy China 2030. Int J Tuberc Lung Dis. 2020;24(3):271-277. doi:10.5588/ijtld.19.0106 32228756

[16] Jiang LL, Liu HY, Ma ZJ, Wang Y. Analysis on current situation and influencing factors of secondhand smoke exposure in indoor public places in Heilongjiang. Article in Chinese. Chinese Journal of Health Education. 2021;37(1):48-51, 56. doi:10.16168/j.cnki.issn.1002-9982.2021.01.010

[17] Sun Y, Chen D, Wang J, et al. Adult secondhand smoke exposure - Shanghai Municipality, 2018. China CDC Wkly. 2020;2(22):399-403. doi:10.46234/ccdcw2020.102 34594666 PMC8428456

[18] Xiong L, Li J. lnvestigation on Second Hand Smoke Exposure of People Aged 15 and Over in Wuhan. Article in Chinese. Chinese Journal of Social Medicine. 2022;39(5):566-570. doi:10.3969/j.issn1673-5625.2022.05.019

[19] Ministry of Commerce of the People’s Republic of China. The Central Committee of the Communist Party of China and the State Council Issued the Outline of the Healthy China 2030 Plan. Website in Chinese. Accessed December 1, 2024. https://m.mofcom.gov.cn/article/b/g/201702/20170202516062.shtml

[20] Wang L, Xu Y, Wu Q. lnvestigating the exposure status of secondhand smoking in adults Of Zhejiang province in 2010 and 2013. Article in Chinese. Chinese Journal of Prevention and Control of Chronic Diseases. 2017;25(8):569-572, 577. doi:10.16386/j.cjpccd.issn.1004-6194.2017.08.003

[21] Xu Y, Liu X, Mei X, Li Y, Wang L, Li J. Current status of awareness rate of tobacco hazard among urban residents in Wuhan City. Article in Chinese. Practical Preventive Medicine. 2021;(8):984-987. doi:10.3969/j.issn.1006-3110.2021.08.023

[22] Rose SW, Emery SL, Ennett S, et al. Public support for family smoking prevention and tobacco control act point-of-sale provisions: results of a national study. Am J Public Health. 2015;105(10):e60-e67. doi:10.2105/AJPH.2015.302751 PMC4566565 26270303

[23] Rhoades RR, Beebe LA, Mushtaq N. Support for local tobacco policy in a preemptive state. Int J Environ Res Public Health. 2019;16(18):3378. doi:10.3390/ijerph16183378 31547351 PMC6766053

[24] Paulik E, Maróti-Nagy A, Nagymajtényi L, Rogers T, Easterling D. Support for population level tobacco control policies in Hungary. Cent Eur J Public Health. 2012;20(1):75-80. doi:10.21101/cejph.a3716 22571024 PMC3821970

[25] World Health Organization. Making Cities Smoke-free. Accessed December 1, 2024. https://www.who.int/publications/i/item/9789241502832

[26] Yang T, Abdullah AS, Li L, et al. Public place smoke-free regulations, secondhand smoke exposure and related beliefs, awareness, attitudes, and practices among Chinese urban residents. Int J Environ Res Public Health. 2013;10(6):2370-2383. doi:10.3390/ijerph10062370 23749054 PMC3717741

[27] United States Public Health Service Office of the Surgeon General; U.S. National Center for Chronic Disease Prevention and Health Promotion Office on Smoking and Health. Smoking Cessation: A Report of the Surgeon General. US Department of Health and Human Services; 2020. Accessed December 1, 2024. https://www.ncbi.nlm.nih.gov/books/NBK555591/

[28] Cook DA, Artino AR Jr. Motivation to learn: an overview of contemporary theories. Med Educ. 2016;50(10):997-1014. doi:10.1111/medu.13074 27628718 PMC5113774

[29] Yang T, Xu X, Rockett IR, Guo W, Zhou H. Effects of household, workplace, and public place smoking restrictions on smoking cessation. Health Place. 2011;17(4):954-960. doi:10.1016/j.healthplace.2011.04.003 21550837

[30] Wulf G, Lewthwaite R. Optimizing performance through intrinsic motivation and attention for learning: the OPTIMAL theory of motor learning. Psychon Bull Rev. 2016;23(5):1382-1414. doi:10.3758/s13423-015-0999-9 26833314

[31] Lin H, Chang C, Liu Z, Zheng Y. Subnational smoke-free laws in China. Tob Induc Dis. 2019;17(November):78. doi:10.18332/tid/112665 31772556 PMC6856824

[32] Yang Y, Li L, Yong HH, et al. Regional differences in awareness of tobacco advertising and promotion in China: findings from the ITC China Survey. Tob Control. 2010;19(2):117-124. doi:10.1136/tc.2009.029868 20008156 PMC2989163

[33] He C, Qu C, Mao T, Yang GP, Li XN. Current situation of the workplace smoking bans and the exposure level of secondhand smoke in Jiangsu Province, 2017. Article in Chinese. Chinese Journal of Health Education. 2019;35(5):400-403, 409. doi:10.16168/j.cnki.issn.1002-9982.2019.05.004

