# Supplementary material for: A cross-sectional study on exposure to secondhand smoke in indoor public places and attitudes of residents towards the smoke control ordinance in public places
Source: Tob Induc Dis. 2024 Dec 27;22:10.18332/tid/196676. doi: 10.18332/tid/196676 (PMC11671798; doi:10.18332/tid/196676)
Supplement: Supplementary file 1 [file TID-22-191-s1.pdf]

Appendix Table 1 Survey sample sizes for each type of establishment, Hangzhou, China, 2022 (N=2746)

Type	N
Government departments	400
Hospitals	400
Training institutes	200
Entertainment venues	400
Restaurants	400
Hotels	400
Shopping malls	400
Universities	140

Appendix Table 2 Demographic characteristics of exposed and non-exposed groups among non-smokers, Hangzhou, China, 2022 (N=2155)

Variable		Exposed group	Non-exposed group	$\chi 2$
**Gender**				2.50
	Male	402	421	
	Female	604	728	
**Age**				11.65*
	15~25	258	332	
	26~35	338	336	

	36~45	202	219	
	46~55	103	108	
	56~65	70	90	
	66~75	35	64	
**Locality type**				3.29
	Urban	174	234	
	Rural	832	915	
**Education level**				24.07***
	Elementary school or less	48	68	
	Junior high school	117	189	
	High school	178	251	
	Junior college/college	591	577	
	Postgraduate or more	72	64	
**Occupation**				16.06**
	Institutionalized personnel	118	94	

	Business units	297	291	
	Students	147	190	
	freelance	163	198	
	Retired	69	96	
	Other	212	280	
**Marital status**				
	Single	423	498	2.02
	Married/Cohabited	555	619	
	Divorced/Separated	21	19	
	Widowed	7	13	
**Chronic diseases**				1.10
	Yes	94	123	
	No	912	1,026	
**Overall**		1,006	1,149	

$^*P < 0.05$, $^{**}P < 0.01$, $^{***}P < 0.001$

Appendix Table 3 Comparison of satisfaction with tobacco control in public places among residents, Hangzhou, China, 2022 (N=2746)

	**Tobacco control publicity campaign**	**$\chi2$**	**Tobacco control supervision and enforcement**	**$\chi2$**	**Smoke-free demonstration for government departments**	**$\chi2$**	**Individual tobacco control behavior**	**$\chi2$**	**Effectiveness of tobacco control in public places**	**$\chi2$**
**Gender**		0.22		4.43*		4.86*		0.47		0.35
Male	855		805		853		912		918	
Female	896		887		938		960		964	
**Age**		34.84***		32.50***		24.91***		30.72***		23.47***
15~25	504		494		519		546		542	
26~35	507		485		524		552		563	
36~45	320		306		327		331		343	
46~55	186		183		192		199		192	
56~65	158		152		156		168		167	
66~75	76		72		73		76		75	
**Locality type**		1.53		2.57		0.08		0.09		0.01
Urban	1,431		1,387		1,454		1,514		1,526	
Rural	320		305		337		358		356	
**Education level**		64.92***		71.85***		49.62***		46.26***		34.22***
Elementary school or less	107		105		106		106		107	
Junior high school	297		290		292		318		311	
High school	430		420		436		447		447	
Junior college/college	843		808		881		914		924	
Postgraduate or more	74		69		76		87		93	

**Occupation**		18.27**		18.42**		11.92*		12.03*		14.41*
Institutionalized personnel	164		161		172		174		177	
Managers and clerks	444		422		465		492		481	
Students	246		237		246		255		253	
freelance	312		302		315		330		345	
Retired	146		136		143		152		149	
Other	439		434		450		469		477	
**Marital status**		12.61**		11.44*		12.44**		9.57		7.70
Single	687		663		705		743		752	
Married/Cohabited	1,014		980		1,036		1,074		1,076	
Divorced/Separated	38		36		36		41		39	
Widowed	12		13		14		14		15	
**Chronic diseases**		0.97		0.44		0.01		2.24		2.21
Yes	201		192		198		218		219	
No	1,550		1,500		1,593		1,654		1,663	
**Tobacco use**		5.40		2.91		1.79		3.30		5.40
No	1,233		1,200		1,284		1,326		1,327	
Yes	400		382		395		414		426	
Ever smoked	118		110		112		132		129	
**Overall**	1,751		1,692		1,791		1,872		1,882	

*$P < 0.05$, **$P < 0.01$, *** $P < 0.001$

© 2024 Wang X. et al.
